# Primary Thromboprophylaxis in Pancreatic Cancer Patients: Why Clinical Practice Guidelines Should Be Implemented

**DOI:** 10.3390/cancers12030618

**Published:** 2020-03-06

**Authors:** Dominique Farge, Barbara Bournet, Thierry Conroy, Eric Vicaut, Janusz Rak, George Zogoulous, Jefferey Barkun, Mehdi Ouaissi, Louis Buscail, Corinne Frere

**Affiliations:** 1 Institut Universitaire d’Hématologie, Université de Paris, EA 3518, F-75010 Paris, France; 2 Assistance Publique Hôpitaux de Paris, Saint-Louis Hospital, Internal Medicine, Autoimmune and Vascular Disease Unit, F-75010 Paris, France; 3 Department of Medicine, McGill University, Montreal, Québec, QC H4A 3J1, Canada; 4 University of Toulouse, F-31059 Toulouse, France; bournet.b@chu-toulouse.fr (B.B.); buscail.l@chu-toulouse.fr (L.B.); 5 CHU de Toulouse, Department of Gastroenterology and Pancreatology, F-31059 Toulouse, France; 6 Institut de Cancérologie de Lorraine, Department of Medical Oncology, Université de Lorraine, APEMAC, EA4360, F-54519 Vandoeuvre-lès-Nancy, France; t.conroy@nancy.unicancer.fr; 7 Department of Biostatistics, Université de Paris, F-75010 Paris, France; eric.vicaut@aphp.fr; 8 Assistance Publique Hôpitaux de Paris, Department of Biostatistics, Fernand Widal Hospital, F-75010 Paris, France; 9 McGill University and the Research Institute of the McGill University Health Centre, Montreal, Québec, QC H4A 3J1, Canada; janusz.rak@mcgill.ca (J.R.);; 10 Department of Digestive, Oncological, Endocrine, and Hepatic Surgery, and Hepatic Transplantation, Trousseau Hospital, CHRU Trousseau, F-37170 Chambray-les-Tours, France; m.ouaissi@chu-tours.fr; 11 Institute of Cardiometabolism and Nutrition, Sorbonne Université, INSERM UMRS_1166, GRC 27 GRECO, F-75013 Paris, France; corinne.frere@aphp.fr; 12 Assistance Publique Hôpitaux de Paris, Department of Haematology, Pitié-Salpêtrière Hospital, F-75013 Paris, France

**Keywords:** pancreatic cancer, venous thromboembolism, thromboprophylaxis, low-molecular weight heparin, direct oral anticoagulant, survival

## Abstract

Exocrine pancreatic ductal adenocarcinoma, simply referred to as pancreatic cancer (PC) has the worst prognosis of any malignancy. Despite recent advances in the use of adjuvant chemotherapy in PC, the prognosis remains poor, with fewer than 8% of patients being alive at 5 years after diagnosis. The prevalence of PC has steadily increased over the past decades, and it is projected to become the second-leading cause of cancer-related death by 2030. In this context, optimizing and integrating supportive care is important to improve quality of life and survival. Venous thromboembolism (VTE) is a common but preventable complication in PC patients. VTE occurs in one out of five PC patients and is associated with significantly reduced progression-free survival and overall survival. The appropriate use of primary thromboprophylaxis can drastically and safely reduce the rates of VTE in PC patients as shown from subgroup analysis of non-PC targeted placebo-controlled randomized trials of cancer patients and from two dedicated controlled randomized trials in locally advanced PC patients receiving chemotherapy. Therefore, primary thromboprophylaxis with a Grade 1B evidence level is recommended in locally advanced PC patients receiving chemotherapy by the International Initiative on Cancer and Thrombosis clinical practice guidelines since 2013. However, its use and potential significant clinical benefit continues to be underrecognized worldwide. This narrative review aims to summarize the main recent advances in the field including on the use of individualized risk assessment models to stratify the risk of VTE in each patient with individual available treatment options.

## 1. Introduction

Exocrine pancreatic ductal adenocarcinoma (PDAC), often referred to simply as pancreatic cancer (PC) is a malignancy with the highest mortality rate of any solid cancer and with a growing incidence, partly due to aging of the population and improvements in diagnostic techniques [1,2]. The global burden of PC as reported by the Global Burden of Disease 2017 PC collaborators showed a 2.3-fold increase in incidence between 1990 and 2017, with 441,000 documented cases in 2017 compared to 196,000 in 1990, and this rise is expected to continue [3]. The prevalence of PC is projected to increase by approximately 40% over the next decade in North America and Europe [4], and it is predicted to become the second-leading cause of cancer-related death by 2030 [5]. However, the factors contributing to the current increasing rate of incidence are not fully understood.

Only 15–20% of PC patients have a potentially resectable tumor at diagnosis, while most patients have locally advanced tumors and over 50% have metastatic disease, due to a lack of early symptoms or available biological markers, with a life expectancy of less than one year [2,6]. Patients undergoing curative resection for PC mostly develop recurrent disease; 69–75% of patients relapse within 2 years and 80–90% relapse within 5 years [7]. Palliative and adjuvant chemotherapy remains the appropriate therapeutic option in unresectable cases and the 5-year survival rate for patients with unresectable tumor is less than 8% [8,9,10]. FOLFIRINOX, which was demonstrated to improve clinical status and survival by Conroy et al. in 2011, is now the current treatment standard for metastatic PC [9]. More recently, Conroy et al. also showed that adjuvant therapy with a modified FOLFIRINOX regimen led to significantly longer survival than gemcitabine (GEM) monotherapy among patients with resected PC [11]. However, despite recent advancements, prognosis remains poor, with few patients surviving to 10 years [7]. In this context, there is a need for optimizing and integrating supportive care in the management of PC patients to improve survival and quality of life. The importance of taking charge of the main physical symptoms related to disease evolution, which include pain, anorexia, depression, duodenal obstruction, ascites and venous thromboembolism (VTE) is well recognized and advocated by disease specialist experts [7]. Although recommended by the International Thrombosis and Cancer Initiative (ITAC) clinical practice guidelines (CPGs) since 2013 [12,13,14] and more recently by the American Society of Clinical Oncology (ASCO) guidelines [15], the use of primary thromboprophylaxis, a supportive treatment with potential significant clinical benefit, continues to be underrecognized [16]. 

## 2. Pancreatic Cancer and Venous Thomboembolism 

### 2.1. Burden of Venous Thromboembolism (VTE) in Pancreatic Cancer: the Highest Incidence of VTE Among All Cancer Types

Cancer is an independent major risk factor for VTE [17,18], the latter occurring in 4% to 20% of all cancer patients [19,20]. The extent of VTE risk is determined by the type of cancer, the stage of the disease, and the location of the tumor [17,21]. PC is the malignancy associated with the highest rate of VTE [19,22]. A strong association between VTE and PC was first reported in an autopsy study of 4258 consecutive necropsies, which documented a VTE event in 56.2% of pancreatic patients compared to 15–25% in other cancer patients [23]. The reported incidence of VTE in PC patients varies from 5% to 41% in retrospective cohorts, depending on the diagnostic methods used (Table 1) [24,25,26,27,28,29,30,31,32,33,34,35,36,37,38,39,40,41,42]. Deep vein thrombosis (DVT) and pulmonary embolism (PE) are the most common VTE events observed [43], but visceral vein thrombosis (VVT), including portal vein thrombosis, splenic vein thrombosis, mesenteric vein thrombosis and hepatic veins thrombosis, accounts for approximatively 30–50% of all reported VTE events [31,38,41,44]. The main risk factors for the onset of VTE in PC patients are advanced or metastatic disease, surgery, or use of chemotherapy [35,39]. The highest incidence of VTE has been reported in a retrospective cohort of PC patients receiving palliative chemotherapy, with a VTE diagnosis in 41.3% of patients [36]. In this study, symptomatic VTE (12.2%) was identified as an independent risk factor for death by multivariate analysis (hazard ratio [HR]: 2.22, 95% CI: 1.05–2.60, *p* < 0.05). We recently investigated the incidence and risk factors for VTE in the BACAP-VTE study, a large prospective multicenter cohort of patients with histologically proven PC. Diagnosis of the index VTE, including DVT, VVT, Catheter-Related Thrombosis (CRT), or PE, was established by the referring physician and based on objective standard routine clinical practice criteria, as previously detailed [41]. During a median follow-up of 19.3 months (95% CI 17.45–22.54), 152 out of 731 (20.79%) patients developed a VTE event. In competing-risk analysis, the cumulative probabilities of VTE were 8.07% (95% CI 6.31–10.29) at 3 months and 19.21% (95% CI 16.27–22.62) at 12 months. The median time from PC diagnosis to VTE was 4.49 months (range 0.8–38.26). The rates of VTE did not differ between patients treated with GEM and those treated with FOLFIRINOX. In a multivariate analysis, primary pancreatic tumor location (isthmus *versus* head, HR 2.06, 95% CI 1.09–3.91, *p* = 0.027) and tumor stage (locally advanced *versus* resectable or borderline, HR 1.66, 95% CI 1.10–2.51, *p* = 0.016 and metastatic *versus* resectable or borderline, HR 2.50, 95% CI 1.64–3.79, *p* < 0.001) were independent predictors for onset of VTE [41]. The PRODIGE 4/ACCORD 11 [9] and PRODIGE 24/ACCORD 24 [11] randomized controlled trials (RCT) reported lower rates of VTE both in metastatic patients (cumulative incidence of grade 3–4 VTE at 6 months, 6.6% in the FOLFIRINOX arm vs. 4.1% in the GEM arm) [9] and in resected pancreatic patients (cumulative incidence of any grade VTE at 6 months, 5.9% in the FOLFIRINOX arm vs. 7.9% in the GEM arm) [11]. Of note, only Common Terminology Criteria for Adverse Events (CTCAE) [45] grade 3 and 4 VTE events were reported in the PRODIGE 24/ACCORD study [11], leading to an underestimate of the overall rate of VTE. In a recent retrospective cohort of 150 PC patients receiving either GEM-based chemotherapy or FOLFIRINOX, there was a 21.4% incidence of incidental and symptomatic VTE (grade 2 or higher) in the FOLFIRINOX group vs. 29.5% in the GEM group, suggesting that patients treated with FOLFIRINOX carry the same risk for VTE as patients treated with GEM-based therapy [38].

### 2.2. Association of VTE with Progression Free Survival and OverAll Survival in Pancreatic Cancer

VTE is the second-leading cause of death after metastasis in cancer patients [46,47]. Patients with cancer who develop VTE have a shorter overall survival compared to those without VTE who have a similar tumor stage and anti-cancer treatment [19]. In a study of 235-149 cancer patients (with 6712 patients with PC) included in the California Cancer Registry, adjusted for age, race, and stage, VTE was a significant predictor of decreased survival during the first year for all cancer types (hazard ratios, 1.6–4.2; *p* < 0.01) and when measured in person-time, the incidence of VTE during the first year after cancer diagnosis, was the highest among patients with metastatic PC (20.0 events per 100 patient-years) [21]. Early retrospective studies assessing the association of VTE with progression-free survival (PFS) and overall survival (OS) in PC patients reported conflicting results. Two monocentric cohorts of PC patients found no difference in OS between patients who developed VTE and those who did not [29,35]. The lack of difference in survival between patients with and without VTE might be explained by patient short life expectancy, since most patients included in both studies had stage III-IV disease. In contrast, several studies have found an association between the onset of VTE and poorer prognosis. In an early monocentric retrospective cohort of 227 patients with unresectable PC, the onset of VTE during chemotherapy was associated with decreased PFS (HR 2.59, 95% CI 1.69–3.97, *p* < 0.0001) and OS (HR 1.64, 95% CI 1.04–2.58, *p* = 0.032) [25]. Similarly, VTE, including VVT, was associated with increased mortality in a small cohort of 135 PC patients. [31] Of note, anticoagulant therapy improved survival in those patients with VTE (HR 0.30, 95% CI 0.12–0.74, *p* = 0.009) [31]. Two retrospective studies focusing on the association of VVT with survival also found an association between the onset of VVT and increased mortality [44,48]. Few studies have investigated the association between early VTE (defined by a VTE at diagnosis or within 30 days after the beginning of palliative chemotherapy) and survival.

**Table 1:** Main studies reporting the rates of venous thromboembolism in pancreatic cancer (PC) patients.

Reference	Study Type	*n*	Study Period or Duration of Follow-Up	Rates of VTE	Type of VTE	Risk Factors for VTE/Survival
Blom et al. 2006 [24]	Cohort Study	202	From January 1990 to December 2000	Incidence rate of VTE: 108.3 per 1000 patient-year (95% CI 64.4–163.8) Overall cumulative incidence of VTE: 94.1 per 1000 patient-year (95% CI 90.9–97.3)	Early VTE: 15 out of 19 cases of VTE occurred in the first 6 months after cancer diagnosis	Risk factors for VTE: Tumor of the corpus (HR 1.9, 95% CI 0.5–6.7) and of the cauda (HR 2.9, 95% CI 1.0–8.5) Chemotherapy (HR 4.8, 95% CI 1.1–20.8) Postoperative period of 30 days (HR 4.5, 95% CI 0.5–40.9) Distant metastases (HR 1.9, 95% CI 0.7–5.1)
Mandala et al. 2007 [25]	Retrospective	227	From December 2001 to December 2004	VTE = 26% (*n* = 59)	VTE at cancer diagnosis in 28 patients (12.3%) VTE occurring during chemotherapy in 15 patients (6.6%)	
Mitry et al. 2007 [26]	Retrospective	90	-	26.7% (*n* = 24)	4 PE, 2 fatal PE	Risk factors for VTE: Use of thromboprophylaxis (HR 0.03, 95% CI 0.003–0.27) Biological inflammatory syndrome (HR 9.0, 95% CI 2.30–34.4) Metastatic disease (HR 4.4, 95% CI 1.1–17.9)
Oh et al. 2008 [27]	Retrospective	75	From June 2003 to December 2005	5.3% (*n* = 4) Incidence rate of VTE: 157 per 1000 patient-year (95% CI 59–418)		
Poruk et al. 2010 [28]	Retrospective	133	-	20% Incidence rate of VTE: 169 per 1000 patient-year (95% CI 109–263)		
Shaib et al. 2010 [29]	Retrospective	201	From July 2003 to December 2008	28.9% (*n* = 58)	Multiple thrombosis: 17.2% (*n* = 10)	
Epstein et al. 2012 [30]	Retrospective	1915	From January 2000 to December 2009	32% (*n* = 650)	Arterial Thrombosis in 1.5% patients (*n* = 30)	
Menapace et al.2011 [31]	Retrospective	135	From 2006 to 2009	34.8% patients (*n* = 47)	12 PE, 28 DVT and 47 VVT Incidental events: 33.3% PE, 21.4% DVT and 100% VVT	Anticoagulants reduced the risk of death by 70% (95% CI 26–88%, *p* = *0*.009)
Afsar et al. 2014 [33]	Retrospective	77	From 2007 to2012	18.1% (*n* = 14)		
Munoz-Martin et al. 2014 [33]	Retrospective	84	From 2008 to 2011	35.7% (*n* = 30)	Multiple thrombosis: 7.1% (*n* = 68) 66 % of the events diagnosed during the first 6 months after diagnosis.	
Larsen et al. 2015 [49]	Prospective	121	Duration of follow-up: 24 months	At the time of cancer diagnosis: 12.4% (*n* = 15) First VTE during follow-up: 20.7% (*n* = 25)		
Ouiassi et al. 2015 [42]	Retrospective	162	Median follow-up of 15 months after diagnosis	17.3% (*n* = 28)		VTE associated with shorter survival (HR 1.995, 95% CI 1.209–3.292)
Krepline et al.2016 [34]	Retrospective	260	From 2009 to 2014	10% (*n* = 26)	All VTE events were incident events: 9 (35%) PE, 9 (35%) DVT, and 8 (31%) VVT	
Lee et al. 2016 [35]	Retrospective	1115	From 2005 to 2010	11.8% (*n* = 132) 2 years cumulative incidence rate of VTE 9.2%	72% of incidental VTE	Major risk factors associated with VTE events: advanced cancer stage, major surgery, and poor performance status
Kruger et al. 2017 [36]	Retrospective	172	From 2002 to 2017	41.3%	50.2% of asymptomatic VTE	
Van Es et al.2017 [37]	Retrospective	178	Median of follow-up of 234 days	12.4% (*n* = 22) - Estimated 2 years overall VTE incidence rate: 13.9% (95% C, 8.3–19.5)	50% of incidental VTE	
Berger et al. 2017 [38]	Retrospective	150	From initiation of first-line treatment until last follow-up or death	25% (*n* = 37)	43.2% of incidental VTE	
Chen et al 2018 [39]	Retrospective	816	From 2010 to 2016	8.0% (*n* = 67)		Leukocyte count > 11,000/μL (HR 1.75, 95% CI 1.07–3.03; *p* = 0.032) Presence of liver metastases (HR 1.65, 95% CI 1.03–3.99; *p* = 0.046)
Kim et al. 2018 [40]	Retrospective	216	From 2005 to 2015	23.6% (*n* = 51)		Risk factors for VTE: Low serum sodium (OR 10.30; 95% CI 1.04–102.47; *p* = 0.047) High Khorana score ≥3 (OR 5.11; 95% CI 1.01–25.84; *p* = 0.049)
Frere et al. 2019 [41]	Prospective	731	From study entry until last follow-up or death Median follow-up of 19.3 months	20.79% (*n* = 152) Cumulative probabilities of VTE: 8.07% (95% CI 6.31–10.29) at 3 months 19.21% (95% CI 16.27–22.62) at 12 months	54% of incidental VTE Median time from PC diagnosis to VTE: 4.49 months (range 0.8–38.26).	Risk factors for VTE:PC tumor location (isthmus versus head, HR 2.06, 95% CI 1.09–3.91, *p* = 0.027) Tumor stage (locally advanced versus resectable or borderline, HR 1.66, 95% CI 1.10–2.51, *p* = 0.016 and metastatic versus resectable or borderline, HR 2.50, 95% CI 1.64–3.79, *p* < 0.001)

Abbreviations: CI, Confidence interval; DVT, deep vein thrombosis; HR, Hazard ratio; OR, odds ratio; PC, pancreatic cancer; PE, pulmonary embolism; VTE, venous thromboembolism; VVT, visceral vein thrombosis.

In 227 unresectable PC patients, Mandala et al. reported that presence of synchronous VTE at cancer diagnosis was associated with a higher probability of not responding to treatment (odds ratio [OR] 2.98, 95% CI 1.42–6.27, *p* = 0.004), but were not associated with PFS or OS on multivariate analysis, while the occurrence of a VTE during chemotherapy was associated with significant shorter PFS (HR 2.59, 95% CI 1.69–3.97, *p*< 0.0001) and OS (HR 1.64, 95%CI 1.04–2.58, *p* = 0.032) [25].

In another monocentric retrospective cohort of 216 metastatic PC patients receiving GEM-based palliative chemotherapy, early VTE occurred in 10.6% patients and was associated with a significantly shorter OS (3.7 months vs. 6.4 months in patients with late VTE or without VTE, *p* = 0.005) [40].

Only two prospective studies have investigated the impact of VTE on survival in PC patients. A small cohort of 121 PC patients reported a significant association between the onset of VTE within the study and shorter OS (median OS 4.4 months vs. 11.9 months in patients without VTE; HR 2.15, 95% CI 1.28–3.60, *p* = 0.004) [49]. In the BACAP-VTE study, patients developing VTE during follow-up had shorter PFS even after adjustment for cancer stage and other risk factors for decreased PFS (6.66 months vs. 9.56 months in patients without VTE; HR 1.74, 95% CI 1.19–2.54, *p* = 0.004). The onset of VTE was also associated with shorter OS, even after adjustment for age, cancer stage and other risk factors for decreased OS (9.13 months vs. 14.55 months in patients without VTE; HR 2.02, 95% CI 1.57–2.60, *p* = 0.004). PC patients who developed VTE after study entry had a higher mortality rate compared to patients who did not develop VTE: 109 out of 152 (72%) patients with VTE died vs. 343 out 531 (65%) patients without VTE (OR 2.88, 95% CI 1.96–4.21, *p* < 0.0001) [41]. These results deserve attention since this association between VTE and mortality suggests that preventing VTE might improve survival in PC patients. 

### 2.3. VTE in Pancreatic Cancer: A Model of Hypercoagulability and the Effects of Heparins

Cancer leads to a hypercoagulable state which confers advantages to cancer cells. This hypercoagulable state is attributed to high expression of tissue factor (TF) and transmembrane proteins (e.g.: PSGL-1, Muc1) by cancer cells [50], leading to thrombin generation and platelet activation and aggregation [51]. Aggregation of blood platelets around cancer cells provides protection from immune responses, and also facilitates circulation of cancer cells in the blood stream and their adhesion at potential sites of metastasis [52,53,54]. In cancer multiple oncogenic events including activation of proto-oncogenes *KRAS* [55], *EGFR*, and inactivation of tumor suppressor genes such as *P53* and *PTEN*, promote TF expression and contribute to other procoagulant changes in the tumor microenvironment [56]. Of note is the fact that in sporadic PC/PDAC over 90% of lesions carry an activating *KRAS* mutation [57] and elevated TF expression is common in advanced stages [58]. Moreover, cancer cells spontaneously release TF-positive microvesicles (MVs) in the circulation [59,60,61]. TF-positive MVs bind to Factor VII (FVII), promoting the activation of the extrinsic pathway and thrombin generation. An additional mechanism of thrombin generation is related to Factor XII activation, which initiates the intrinsic coagulation pathway. Moreover, Plasminogen Activator Inhibitor 1 (PAI-1) can be released by pancreatic tumor cells, as well as by activated platelets [62]. TF initiates angiogenesis via both a) a clotting-dependent mechanism where thrombin formation and fibrin deposition support angiogenesis, and TF induces VEGF expression, and b) a clotting-independent mechanism in which TF-FVIIa complex activates pro-angiogenic protease-activated receptor 2 (PAR-2). In addition, an alternatively spliced TF (asTF, soluble variant of TF) is expressed in PDAC as opposed to normal pancreas, and stimulates angiogenesis independently of FVIIa [63,64]. Unruh et al. demonstrated that asTF binds β1-integrins on the surface of PDAC cells and also on microvascular endothelial cells [63,64], thereby promoting tumor growth, metastatic dissemination, and monocyte recruitment to the stroma through an autocrine paracrine manner [64]. Overall, TF overexpression by the PC cells which induces thrombin generation and platelet activation all directly contribute to cancer progression and dissemination [65]. Whether and how preceding intermittent inflammation and PC-associated desmoplasia contribute to these events remain of great interest [57]. 

Circulating extracellular MVs derived from cancer-cells contribute to hypercoagulability and to metastatic invasion. Experimental data have shown that MVs released by cultured PC cells exhibit TF-dependent procoagulant activity [66]. In a mouse model of PC, cancer cell-derived MVs expressing TF accumulate at sites of endothelial injury in a *p*-selectin-dependent manner [67]. 

Several publications demonstrate the presence of TF-bearing MVs in patients with cancer [68,69,70,71,72,73,74,75]. In PC patients specifically, around 50% of TF-positive MVs detected in platelet-poor plasma are also positive for MUC-1 antigen, suggesting that they are derived from the underlying malignancy. TF-positive MVs are highly procoagulant [69]. A retrospective study of 117 patients with pancreatic or biliary cancer (68% of PC) reported a 44.4% rate of VTE. In these patients, elevated TF levels were significantly associated with VTE events (*p* = 0.04), and with decreased overall survival (HR 1.05; *p* = 0.01) [70]. A first prospective study suggested that MVs-TF activity may be predictive of VTE in PC patients [71]. In a cohort study of 73 PDAC patients, elevated MVs-TF activity was present only in patients with poorly differentiated metastatic, unresectable tumors and correlated with CA 19–9 and D-dimer levels [73]. In a prospective cohort study on 79 PDAC patients, MVs-TF activity did not correlate with the intensity of TF expression in adenocarcinoma cells but to the number of TF-positive macrophages in the surrounding stroma [74]. More recently, Faille et al. showed that MVs-TF activity was predictive of VTE in 48 PDAC patients [75]. Both D-dimers and MVs-TF activity were associated with the occurrence of VTE [75].

Many studies have analyzed the effect of heparins and of low molecular weight heparin (LMWH) on tumor progression, metastasis formation, and angiogenesis [76,77]. In addition to its action on the coagulation cascade, heparin also inhibits the binding of P-selectin to its ligands [78], which is involved in hypercoagulability and metastasis process. Heparins as well as heparan sulfate (HS) belong to the glycosaminoglycan family and bind antithrombin via a pentasaccharide sequence. HS are key components of the extracellular matrix (ECM). Heparanase, which is overexpressed in PC, acts by cleaving heparan sulfate side chains from proteoglycans, contributing to ECM disruption and vascular endothelial growth factor A (VEGF-A) and fibroblast growth factor 2 (FGF-2) release [51]. In addition, heparanase has a non-enzymatic pro-coagulant activity in which removal of glycocalyces containing tissue factor pathway inhibitor (TFPI) enhances TF activity [79]. By inhibiting heparanase, heparins may potentially inhibit tumor growth [80].

LMWHs can also contribute to the inhibition of cancer progression. LMWHs were shown to inhibit P-and L-selectin as well as integrin-mediated formation of tumor thrombi, and to alter tumor neo-angiogenesis. This effect is primarily based on their ability to induce the prolonged release of TFPI from binding sites on endothelial cells [81,82]. TFPI acts by inhibiting TF-FVIIa complex, leading to activation of Factor X, inhibition of thrombin generation and PAR-2 activation, and disruption of pro-angiogenic signaling [83,84]. Thus, anticoagulants could possess both biological and antithrombotic activities in cancer albeit possibly exerted through different mechanisms. 

## 3. Risk Assessment Models (RAM) for Prediction of VTE in Patients with PC

Risk assessment models (RAM) have been developed to help identify cancer patients at high risk of VTE who may benefit from primary thromboprophylaxis (Table 2). Nonetheless, VTE risk factors vary according to cancer type and during the course of malignancy, from diagnosis through treatment, metastasis, and end-of-life care. Therefore, repeated individual risk assessments are important.

The most widely used RAM for VTE prediction in ambulatory cancer patients is the Khorana score (KS). This score was developed in a prospective derivation cohort of 2701 cancer outpatients in the United States more than ten years ago and validated in an independent cohort of 1365 patients from the same study [85]. The KS assigns different points to five clinical and pre-chemotherapy laboratory parameters, namely: primary tumor site (+2 points for PC and gastric cancer), platelet count ≥350 × 10^9^·dL^−1^ (+1 point), hemoglobin concentration ≤ 10 g.dL^−1^ or use of erythropoiesis-stimulating agents (+1 point), leukocyte count ≥11 × 10^9^·L^−1^ (+1 point), and a BMI ≥ 35 kg/m^2^ (+1 point). The KS discriminates three groups of patients according to risk of VTE: a low-risk group (score of 0), an intermediate-risk group (score of 1–2) and a high-risk group (score ≥3). Several small retrospective studies in PC patients undergoing chemotherapy found no difference in the rates of VTE between intermediate and high-risk patients, as estimated by the Khorana score (Table 3) [33,36,37,38,86]. In the BACAP-VTE study, the KS did not discriminate between patients with intermediate vs. high VTE risk scores [41]. However, all PC patients have a sum score ≧ 2 and being subsequently classified as at least intermediate-risk or high risk of VTE should be considered for prophylaxis. Other RAMs, such as the Vienna modification of the Khorana score (addition of biomarkers D-dimer and soluble P-selectin) [87], the PROTECHT score (addition of GEM and platinum-based chemotherapy), [88] and the CONKO score (addition of WHO performance status) [86] have been developed. However, none of these scores have been externally validated in PC patients.

The ONKOTEV score [89] was developed using a large multicenter prospective cohort of 843 cancers patients. Overall, 73 (8.6%) VTE events occurred during a median follow-up of 8.3 months. In a multivariate analysis, the presence of a metastatic disease, the compression of vascular/lymphatic structures by the tumor, a history of previous VTE, and a KS ≥2 were significantly associated with the risk of VTE. The resulting ONKOTEV score assigns one point to each of these four variables, and according to a sum score of 0, 1, 2, or ≥ 2 points patients are classified as being at “score = 0”, “score = 1”, “score = 2”, or “score > 2”, respectively. In the development cohort, patients with “score = 0” (37%) had a cumulative probability of VTE at 12 months of 3.69%, compared to 9.74% for patients with “score = 1” (43.1%), 19.39% for patients with “score = 2” (9.2%), and 33.87% for patients with “score > 2” (6.3%). As expected, the ONKOTEV score demonstrated a significantly higher predictive power than the KS in the same cohort. However, overfitting of the ONKOTEV model is likely and these results should be interpreted with caution. This model was recently externally validated in a retrospective single-center cohort of 165 PC patients [90]. Cumulative incidence of VTE was 3.3%, 12.7%, 50.9%, and 82.4% for patients with ONKOTEV scores of 0 (18.2% of the overall population), 1 (38.2% of the overall population), 2 (33.3% of the overall population), and >2 (10.3% of the overall population), respectively [90]. This study has several limitations including its retrospective and single-center design, and the inclusion of patients with VTE at cancer diagnosis and deserve further confirmation in prospective cohorts of ambulatory PC patients.

**Table 2:** Risk assessment models that have been evaluated in pancreatic cancer patients.

**KHORANA SCORE [85]**	
Very high-risk tumors (stomach, pancreas)	**+2**
High risk tumors (lung, gynecologic, genitourinary excluding prostate)	**+1**
Hemoglobin <10 g/dl or erythropoietin stimulating agents	**+1**
White blood cell count >11 × 10^9^/L	**+1**
Platelet count ≥ 350 × 10^9^/L	**+1**
BMI >35 kg/m^2^	**+1**
A score of 0 = low-risk category A score of 1–2 = intermediate-risk category A score of >2 = very high-risk category	
**ONKOTEV SCORE [89]**	
Khorana score of >2	**+1**
Previous venous thromboembolism	**+1**
Metastatic disease	**+1**
Vascular/lymphatic macroscopic compression	**+1**
Total ONKOTEV score	**4**

Abbreviations: BMI = body mass index.

**Table 3:** Studies assessing the predictive values of risk assessment models in pancreatic cancer patients.

Reference	Type	RAMs	Study Population, *n*	VTE Screening at Study Entry	Median Follow Up (Months)	Number of Patients in Each Group	Patients with VTE During the Total Follow-Up, *n* (%)	Rates of VTE
Pelzer et al. 2013 [86]	Retrospective	Khorana score	144	No	12	Intermediate risk: 38% High risk: 62%	21 (14.6%)	At 6 months: Intermediate risk: 7.2% High risk: 19.1%
Munoz-Martin et al. 2014 [33]	Retrospective	Khorana score	73	No	9.5	Intermediate risk: 51% High risk: 49%	22 (30.1%)	At 6 months: Intermediate risk: 10.8% High risk: 27.8%
Van Es et al. 2017 [37]	Retrospective	Khorana score	147	No	7.7	Intermediate risk: 31% High risk: 69%	20(13.6%)	At 6 months: Intermediate risk: 8.9% High risk: 8.7%
Kruger et al. 2017 [36]	Retrospective	Khorana score	111	No	9.2	Intermediate risk: 62% High risk: 38%	16 (14.4%)	At 6 months: Intermediate risk: 8.7% High risk: 11.9%
Berger et al. 2017 [38]	Retrospective	Khorana score	150	No	NS	Intermediate risk: 58% High risk: 42%	37 (24.7%)	NS During the total follow-up: no difference between groups (*p* = 0.44)
Godinho et al. 2019 [90]	Retrospective	Onkotev score	165	no	6.3	Score 0: 18.2% Score 1: 38.2% Score 2: 33.3% Score ≥3: 10.3%	51 (31%)	During the total follow-up: Score 0: < 10% Score 1: <10% Score 2: 41.8% Score ≥3: 70.6%
Frere et al. 2019 [41]	Prospective	Khorana score	731	Yes	19.3	Intermediate risk: 73% High risk: 27%	152 (20.1%)	NS During the total follow-up: Intermediate risk: 21% High risk: 18% Intermediate vs. high risk: HR 0.83 (95% CI 0.56–1.23), *p* = 0.363

Abbreviations: NS, not specified; RAM, risk assessment model; VTE, venous thromboembolism.

## 4. Studies Assessing the Benefit of Anticoagulants in Pancreatic Cancer Patients

### 4.1. Primary Thromboprophylaxis in Ambulatory Pancreatic Cancer Patients

#### 4.1.1. Primary Thromboprophylaxis with LMWH in Cancer Patients

Several RCTs have assessed the efficacy and safety of LMWH for primary thromboprophylaxis in patients with different cancers treated with chemotherapy. The PROTECHT [91] and SAVE-ONCO [92] trials enrolled more than 4000 patients with non-selected solid cancers, but few data were generated from the subgroup analyses of PC patients (Table 4).

In PROTECHT [91], 1150 patients with different cancers were randomized to receive either nadroparin (3800 IU once daily) or placebo for the duration of chemotherapy, up to a maximum of 4 months. A significant reduction in the rate of VTE was observed in the nadroparin arm (2.0% vs. 3.9% in the placebo arm, *p* = 0.02) without difference in major bleeding (0.7% in the nadroparin arm vs. 0 in the placebo arm, *p* = 0.18). Only 53 out of 1150 (4.7%) patients included in PROTECHT had PC, and the rates of VTE did not differ between the placebo and nadroparin treatment arms in this subgroup of PC patients (*p* = 0.755). This lack of difference might be related to the small number of PC patients included in PROTECHT.

In SAVE-ONCO [92], 3212 patients with metastatic or locally advanced cancers beginning a course of chemotherapy were randomized to receive either semuloparin (20 mg once daily) or placebo for the duration of chemotherapy. In the overall population, a significant reduction in the rates of VTE was observed in the semuloparin arm (1.2% vs. 3.4% in the placebo arm; HR 0.36, 95% CI 0.21–0.60; *p* < 0.001), and there was no difference in major bleeding (1.2% vs. 1.1% in the placebo arm; HR 1.05, 95% CI 0.55–1.99; *p* = ns). Two hundred fifty four out of 3212 (7.9%) patients had a PC in SAVE-ONCO. In this PC patient subgroup, a significant reduction in the rates of VTE was observed in the semuloparin arm (2.4% vs. 10.9% in the placebo arm; HR 0.22, 95 CI 0.06–0.76; *p* = 0.015). This subgroup effect was not statistically significant different, from the overall effect in the overall population.

#### 4.1.2. Primary Thromboprophylaxis with LMWHs in Pancreatic Cancer Patients

Two dedicated RCTs evaluated the efficacy and safety of primary thromboprophylaxis with LMWH in patients with advanced PC receiving chemotherapy (Table 4) [93,94]. In total, these two studies enrolled more than 400 PC patients. 

The phase 2b FRAGEM trial randomized 123 advanced PC patients to receive either GEM with weight-adjusted dalteparin (GEM-WAD, dalteparin 200 IU/kg daily during 4 weeks, then 150 IU/kg daily) for 12 weeks or GEM alone [93]. The primary end point was the occurrence of all-type VTE (symptomatic or incidentally diagnosed). Addition of weight-adjusted dalteparin reduced the rate of VTE from 23% to 3.4% during the treatment period (RR 0.145, 95% CI 0.035–0.612, *p* = 0.002) and from 28% to 12% during the entire follow-up period (RR 0.42, 95% CI 0.19–0.94, *p* = 0.039). VTE-related deaths were observed in 5 (8.3%) patients in the GEM alone arm compared to 0 patients in the GEM with weight-adjusted dalteparin arm (RR 0.092, 95% CI 0.005–1.635, *p* = 0.057). The rates of major bleeding events were low in both arms (3.4% in the GEM-WAD arm vs. 3.2% in the GEM arm), but there was a higher incidence of trivial bleeding (skin bruising, minor epistaxis) in the GEM-WAD arm (9% vs. 3% in the GEM arm) [93].

The prospective, open-label, multicenter phase 2b PROSPECT-CONKO 004 study randomized 312 advanced PC patients to receive enoxaparin during the first 12 weeks of chemotherapy (1 mg/kg daily for the first 3 months, then 40 mg daily, *n* = 160) or chemotherapy alone (i.e., single-agent GEM or an intensified regimen including fluorouracil and folinic acid, depending on performance status and renal function, *n* = 152) [94]. The primary end point was the first event rate of symptomatic VTE within 3 months. Asymptomatic VTE events found on routine imaging during the study were excluded from the analysis. Enoxaparin reduced the cumulative incidence rate of symptomatic VTE from 10.2% to 1.3% within the first 3 months (HR 0.12, 95% CI 0.03–0.52, *p* = 0.001) and from 15.1% to 6.4% during the entire follow-up period (HR 0.40, 95% CI 0.19–0.83, *p* = 0.01), without an overall difference in major bleeding (8.3% in the enoxaparin arm vs. 6.9% in the control arm, HR 1.23, 95% CI 0.54–2.79, *p* = 0.63). Three fatal bleeding events occurred in the overall population, including one fatal bleed from esophageal varices in the enoxaparin arm and two fatal bleeds from fulminant cancer ulceration in the duodenum in the control arm. There was no significant difference in PFS (HR, 1.06; 95% CI, 0.84 to 1.32; *p* = 0.64) or OS (HR, 1.01; 95% CI, 0.87 to 1.38; *p* = 0.44).

A recent meta-analysis pooling the results from both the FRAGEM [93] and PROSPECT-CONKO 004 [94] trials reported a significant reduction in crude rates of VTE in advanced PC patients receiving LMWH compared to control (2.1% vs. 11.2%, RR 0.18, 95% CI 0.08–0.40), corresponding to a 82% relative risk reduction, without difference in the rate of bleeding events (4.1% vs. 3.3%, RR 1.25, 95% CI 0.48–3.3) [95]. While standard prophylactic doses of LMWH were used in PROTECHT [91] and SAVE-ONCO [92] trials, dalteparin was administered at therapeutic doses in the FRAGEM trial, [93] and enoxaparin was administered at supra-prophylactic doses in the PROSPECT-CONKO 004 trial, [94] suggesting that PC patients might require higher than standard prophylactic doses of anticoagulant for effective VTE prophylaxis.

#### 4.1.3. Direct Oral Anticoagulants (DOAC) As Primary Thromboprophylaxis in Various Cancers, with PC as A Subgroup

Despite limited data on their efficacy and safety in this setting, there is growing interest in the potential role of DOACs for thromboprophylaxis in patients with cancer. Two recent randomized controlled trials evaluated the efficacy and safety of primary thromboprophylaxis with DOACs in various cancers with different inclusion criteria and primary endpoints (Table 4).

The double-blind placebo-controlled CASSINI trial randomized 841 cancer patients initiating chemotherapy at intermediate-to-high risk of VTE (as defined by a Khorana score ≥2) to receive either primary prophylaxis with rivaroxaban (10 mg once daily) or placebo for up to 6 months. [96] Four hundred eight patients (54.5%) had stage IV disease at enrollment. Screening ultrasound was performed at baseline and every 8 weeks during the follow-up period. The primary efficacy endpoint was a composite of symptomatic DVT, asymptomatic proximal DVT, any PE and VTE-related death within the first 180 days after randomization. During the entire follow-up, there was no statistically significant difference in the primary end point between the two arms: 6.0% in the rivaroxaban arm vs. 8.8% in the placebo arm (HR 0.66, 95% CI 0.40–1.09; *p* = 0.10). However, during the on-treatment period, patients treated with rivaroxaban experienced fewer VTE events compared to those receiving placebo (2.6% vs. 6.4%, HR 0.40, 95% CI 0.20–0.80). There was no difference in major bleeding between the two groups (HR 1.96, 95% CI 0.59–6.49). In a prespecified subgroup analysis of PC patients included in CASSINI (*n* = 273, 32.6%), the primary composite endpoint occurred in five out of 135 (3.7%) PC patients in the rivaroxaban arm compared to 14 out of 138 (10.1%) patients in the placebo arm (HR 0.35, 95% CI 0.13–0.97) during intervention. In PC patients, there was no difference in major bleeding between the two groups (1.5% in the rivaroxaban arm vs. 2.3% in the placebo arm) [97].

The Double-Blind Placebo-Controlled Phase 3 AVERT Trial [98] randomized 574 ambulatory cancer patients initiating chemotherapy at intermediate-to-high risk of VTE (as defined by a Khorana score ≥2) to receive either primary prophylaxis with apixaban (2.5 mg twice daily) or a placebo for up to 6 months. Five hundred and sixty-three patients were included in the modified intention-to-treat analysis. One hundred forty (24.8%) patients had metastatic disease at enrollment and 77 (13.8%) patients had PC. Screening ultrasound was not performed at baseline, nor during the follow-up period. The primary outcome was the occurrence of objectively documented major VTE (proximal DVT or PE) within the first 180 days after randomization. During the on-treatment period, patients receiving apixaban had a significant lower risk of VTE (1% vs. 7.3% in the placebo arm; HR 0.14, 95% CI 0.05–0.42, *p* < 0.001) with no difference in major bleeding (2.1% in the apixaban arm vs. 1.1% in the placebo arm; HR 1.89, 95% CI 0.39–9.24). 

**Table 4:** Studies assessing the clinical benefit of anticoagulants for the prevention of venous thromboembolism in ambulatory pancreatic cancer (PC) patients.

Reference Study Design	Number of Patients Analyzed	Follow-Up	Population	Intervention	VTE Incidence	Safety	Survival
**PROTECHT** Agnelli et al. 2009 [91] Randomized, placebo-controlled, double-blind, multicenter study	Overall population Arm A: 769 patients Arm B: 381 patients PC subgroup Arm A: 36 patients Arm B: 17 patients	120 days	Ambulatory patients >18 years on chemotherapy with metastatic or locally advanced lung, gastrointestinal, breast, ovarian, or head and neck cancer	Arm A: nadroparin 3800 IU/day Arm B: placebo For duration of chemotherapy (up to 4 months maximum)	**Overall population** Arm A: 11/769 (1.4%) Arm B: 11/381 (2.9%) *p* = 0.02 **PC subgroup** Arm A: 3/36 (8.3%) Arm B: 1/17 (5.9%) *p* = 0.755	**Overall population** Major bleeding Arm A: 5/769 (0.7%) Arm B: 0/381 *p* = 0.18 Minor bleeding Arm A: 57/769 (7.4%) Arm B: 30/381 (7.9%) *p* = ns **PC subgroup** NS	**Overall population** Arm A: 33/769 (4∙3%) Arm B: 16/381 (4.2%) *p* = *ns* **PC subgroup** NS
**SAVE ONCO** Agnelli et al. 2012 [92] Randomized, placebo-controlled, double-blind, multicenter study	Overall Population Arm A: 1608 patients Arm B: 1604 patients PC subgroup Arm A: 126 patients Arm B: 128 patients	3 months	Patients with metastatic or locally advancedlung, pancreatic, gastric, colorectal, bladder, and ovarian cancer beginning to receive a course of chemotherapy	Arm A: Semuloparin, 20 mg/day Arm B: placebo For duration of chemotherapy (median: 3.5 months)	**Overall population** Arm A: 20/1608 (1.2%) Arm B: 55/1064 (1.2%) HR 0.36 (95%CI 0.21–0.60) *p* < 0.001 **PC subgroup** Arm A: 3/126 (2.4%) Arm B: 14/128 (10.9% HR 0.22 (95%CI 0.06–0.76) *p* = 0.015	**Overall population** Major bleeding Arm A: 19/1589 (1.2%) Arm B: 18/1583 (1.1%) OR 1.05 (95% CI 0.55–2.04) CRNMB Arm A: 26/1589 (2.8%) Arm B: 14/1583 (0.9%) OR 1.86 (95% CI 0.98–3.68) **PC subgroup** NS	NS
**FRAGEM** Marayevas et al. 2012 [93] Randomized, controlled Phase 2b study	Arm A: 59 patients Arm B: 62 patients	3 months	Patients aged 18 years or older Histologically/cytologically confirmed advanced ormetastatic pancreatic cancer Karnofsky performance status (KPS): 60–100	Arm A: Gemcitabine + Dalteparin 200 IU/kg sc, od, for 4 weeks, followed by a step‑down regimen to 150 IU/kg for a further 8 weeks) Arm B: Gemcitabine alone For up to 12 weeks	**At 3 months** Arm A: 2/59(3%) Arm B: 14/62 (23%) RR 0.145 (95% CI 0.035–0.612) *p* = 0.002 **Entire study** Arm A: 7/59 (12%) Arm B: 17/62 (28%) RR 0.419 (95%CI 0.187–0.935) *p* = 0.039	ISTH severe Arm A: 2/59 (3%) Arm B: 2/62 (3%) ISTH non severe Arm A: 5/59 (9%) Arm B: 2/62 (3%)	**Arm A:** 8.7 months **Arm B:** 9.7 months
**CONKO-0004** Pelzer et al. 2015 [94] Prospective, open label, randomized, multicenter and group-sequential 2b trial	Arm A: 160 patients Arm B: 152 patients	3 months	Patients with histologically proven advanced pancreatic cancer were randomly assigned to ambulant first-line chemotherapy	**Arm A:** Enoxaparin 1 mg/kg/day **Arm B:** no enoxaparin	**At 3 months** **Arm A:** 2/160 (1.25%) **Arm B:** 15/152 (9.8%) HR 0.12 (95%CI 0.03–0.52) *p* = 0.001 **Cumulative incidence rates** **Arm A:** 6.4% **Arm B:** 15.1% HR 0.40 (95% CI 0.19–0.83) *p* = 0.01	**Cumulative incidence rates** **Of major bleeding** **Arm A:** 8.3% **Arm B:** 6.9% HR 1.23 (95% CI 0.54–2.79) *p* = 0.63	**Arm A:** 8.2 months **Arm B:** 8.51 months HR 1.01 (95% CI 0.87–1.38) *p* = 0.44
**CASSINI** Khorana et al. 2019 [96] Double-blind, randomized, placebo-controlled, parallel-group, multicenter study	Overall population Arm A: 420 patients Arm B: 404 patients PC patients Arm A: 135 patients Arm B: 138 patients	6 months	Adult ambulatory patients with various cancers initiating a new systemic regimen and at increased risk for VTE (defined as Khorana score ≥ 2).	**Arm A:** rivaroxaban 10 mg once daily up to day 180 **Arm B:** placebo up to day 180	**Overall population** **VTE at 6 months** **Arm A:** 25/420 (5.95%) **Arm B:** 37/421 patients (8.79%) HR 0.66 (95% CI 0.40–1.09) *p* = 0.101 NNT = 35 **VTE during the on-treatment period** **Arm A:** 11/420 (2.62%) **Arm B:** 27/421 (6.41%) HR 0.40 (95% CI 0.20–0.80) *p* = 0.007 NNT = 26 **PC subgroup** **Composite of VTE and death from VTE** **Arm A:** 5/135 (3.7%) **Arm B:** 14/138 (10.1%) HR 0.35 (95% CI 0.130–0.97) *p* = 0.03	**Overall population** **Major bleeding** **Arm A:** 8/405 (1.98%) **Arm B:** 4/404 (0.99%) HR 1.96 (95% CI 0.59–6.49) *p* = 0.265 NNH = 101 **Clinically relevant non-major bleeding** **Arm A:** 2.72% **Arm B:** 1.98% HR 1.96 (95% CI, 0.59–6.49) *p* = 0.265 NNH = 101 **PC subgroup** **Major bleeding** **Arm A:** 2/135 (1.5%) **Arm B:** 3/138 (2.3%)	**Overall population** **All-cause mortality** **Arm A:** 20.0% **Arm B:** 23.8% HR, 0.83, 95% CI 0.62–1.11 *p* = 0.213. **PC subgroup** NS
**AVERT** Carrier et al. 2019 [98] Double-blind, randomized, placebo-controlled, multicenter study	Overall population Arm A: 288 patients Arm B: 275 patients PC patients: 77	6 months	Ambulatory cancer patients receiving chemotherapy who are at high-risk for VTE (as defined by a Khorana score of ≥2)	**Arm A:** apixaban 2.5 mg twice daily up to day 180 **Arm B:** placebo up to day 180	**Overall population** **VTE at 6 months** **Arm A:** 12/288 (4.2%) **Arm B:** 28/275 (10.2%) HR 0.41 (95% CI 0.26–0.65) *p* < 0.001 **PC subgroup** NS	**Overall population** **Major bleeding** **Arm A:** 10/288(3.5%) **Arm B:** 5/275(1.8%) HR 2.00 (95% CI 1.01–3.95) *p* = 0.046 **Clinically relevant non-major bleeding** **Arm A:** 21/288 (7.3%) **Arm B:** 15/276 (5.5%) HR, 1.28; 95% CI, 0.89–1.84 **PC subgroup** NS	**All-cause mortality** **Arm A:** 35/288 (12.2%) **Arm B:** 27/275 (9.8%) HR 1.29 (95% CI 0.98–1.71) *p* = ns **PC subgroup** NS
Ramathan et al. 2018 [99] Open-label multicenter phase 2	Arm A: 18 patients Arm B: 16 patients	Median of 8 weeks	Locally advanced ductal adenocarcinoma of the pancreas diagnosed ≤6 months prior to enrollment	**Arm A:** Gemcitabine +PCI-27483 1.2 mg/kg/bid **Arm B:** Gemcitabine alone	**VTE (any grade)** **Arm A:** 10/18 (56%) **Arm B:** 3/16 (19%)	**Bleeding (any grade)** **Arm A:** 1/18 (6%) **Arm B:** 2/16 (13%)	**Arm A:** 5.7 months **Arm B:** 5.6 months

Abbreviations: CI, confidence interval; CRNMB, clinically relevant non major bleeding; HR, hazard ratio; OR, odds ratio; NNH, number needed to harm; NNT, number needed to treat; NS, not specified; PC, pancreatic cancer; RR, relative risk; VTE, venous thromboembolism.

During the entire follow-up, patients receiving apixaban experienced fewer VTE events compared to those receiving placebo (4.2% vs. 10.2%; HR 0.41, 95% CI 0.26–0.65, *p* < 0.001). In the modified intention-to-treat analysis, patients receiving apixaban had a significant higher risk of major bleeding (3.5% vs. 1.8% in the placebo arm; HR 2.00, 95% CI 1.01–3.95; *p* = 0.046). Results were not reported separately for the subgroup of PC patients. 

The percentage of patients who prematurely discontinued the trial regimen was relatively high in both trials (47% in CASSINI and 38% in AVERT) and there was no significant difference in overall survival between patients receiving DOAC or placebo. 

In an updated meta-analysis pooling the results from FRAGEM, [93] PROSPECT-CONKO 004 [94] and from subgroups of PC patients included in PROTECHT [91], SAVE-ONCO [92] and CASSINI, [96] PC patients receiving primary thromboprophylaxis with either LMWH or a DOAC had significantly lower rates of VTE compared to controls (5.43% vs. 12.07%, RR 0.44, 95% CI 0.29–0.70), with a risk difference of −0.06 (95% CI −0.11–0.01, *p* = 0.01) with no difference in the rate of major bleeding between the two groups (4.11% vs. 3.27%) [100]. However, pooling subgroup analyses of RCTs is prone to biased results and these results should be interpreted with caution.

Compared to LMWH, DOACs have the advantage of being orally administered at fixed doses. However, there are some limitations for their use in cancer patients that should be taken in consideration. Certain patient characteristics (e.g.: weight and age) and comorbidities (e.g.: renal or hepatic impairment), as well as potential drug-drug interactions may affect anticoagulant pharmacokinetics and result in over or under-coagulating [101,102,103]. Vomiting and diarrhea, common side effects of cancer treatment, can also limit drug absorption in these patients. Finally, DOACs have been associated with an increased risk of GI bleeding, particularly in cancers of the upper GI tract. In each case, full consideration of the appropriate balance of benefits and harms is warranted.

### 4.2. Anticoagulants as Adjuvant Treatment to Improve Survival in Pancreatic Cancer Patients

Several studies evaluated the hypothesis that targeted inhibition of the coagulation cascade might improve survival in cancer patients. However, few data were obtained from PC patients due to their short life expectancy. 

The FAMOUS trial [104] randomized 385 cancer patients to receive either dalteparin (5000 IU daily) or placebo for 1 year. One year after randomization, OS was 46% in patients receiving dalteparin compared to 41% in patients receiving placebo (*p* = 0.19). Thirty eight (10%) PC patients were included in the study, but results were not reported for this subgroup [104].

The MALT trial [105] assessed the effect of nadroparin compared to placebo for 6 weeks on survival in 302 patients with advanced cancer without VTE. In the intention-to-treat population, the median survival was significantly longer in the nadroparin group (8.0 months vs. 6.6 months in the placebo group; HR 0.75, 95% CI 0.59–0.96; *p* = 0.021), even after adjustment for WHO performance status, concomitant treatment, and type and histology of cancer (HR 0.76, 95% CI 0.58–0.99). In a pre-specified subgroup of patients with a life expectancy longer than 6 months at enrollment, the median survival was 15.4 months in the nadroparin group compared to 9.4 to months in the placebo group (HR 0.64, 95% CI 0.45–0.90; *p* = 0.01). These results are difficult to extrapolate to PC patients since only 18 out of 302 (6%) patients included in the MALT trial had PC [105].

In a multicenter, open-label, randomized controlled trial, 503 patients with non-small-cell lung cancer, hormone-refractory prostate cancer, or locally advanced PC received either nadroparin for 6 weeks (2 weeks at therapeutic dose, and 4 weeks at half therapeutic dose) in addition to their cancer treatment, or no nadroparin. One hundred thirty four out of 503 (27%) patients had PC. In PC patients, the mortality rate did not differ between the two study arms (79% in the nadroparin arm vs. 73.6% in the control arm; HR 1.14, 95% CI 0.77–1.68; *p* = 0.53). The median survival was 8.0 months in the nadroparin arm compared to 10.4 months in the control arm (*p* = ns) [106].

Finally, a non-randomized trial reported that the use of nadroparin improved survival in 69 consecutive patients with advanced pancreatic ductal adenocarcinoma treated with GEM plus cisplatin every 21 days with or without nadroparin until disease progression. The overall response rate on PFS was 58.8% with nadroparin compared to 12.1% without nadroparin (*p* = 0.0001). Patients receiving nadroparin had longer median time to progression and survival compared to those without (7.3 vs. 4.0 months, *p* = 0.0001 and 13.0 vs. 5.5 months, *p* = 0.0001, respectively) [107]. 

Survival was a secondary efficacy end point in FRAGEM [93] and PROSPECT-CONKO 004 [94], but despite established association between VTE and mortality in PC patients, both studies failed to demonstrate a benefit of LMWH on overall survival (Table 4). This lack of difference between the LMWH and placebo arms might be related to the short life expectancy of PC patients included in these studies [108], or might suggest that the activities driving VTE and progression are not equally susceptible to LMWH. It may well be that clinical VTE per se is not the sole determinant of survival and neither could be FXa and FIIa since TF activities unrelated to those e.g., PAR2 would not be altered by LMWH.

A recent phase 2 study evaluated the safety and efficacy of PCI-27483, a reversible small-molecule inhibitor of activated factor VII. This study randomized 34 patients with metastatic or locally advanced PC to receive PCI-27483-GEM (*n* = 18) or GEM alone (*n* = 16). OS did not significantly differ between patients treated with PCI-27483- GEM and those with GEM alone but there was a nonsignificant trend toward longer PFS in patients receiving PCI-27483- GEM compared to those receiving GEM alone (PFS: 3.7 months vs. 1.9 months; HR 0.62; *p* = 0.307) [99]. There was no difference in the rates of grade ≥3 bleeding between the two arms and there was a trend toward lower rates of VTE in the PCI-27483- GEM arm (6% vs. 13% in the GEM arm). Overall, there is yet no evidence that points towards any survival benefit of anticoagulants as adjuvant treatment in PC patients.

## 5. Current Guidelines for VTE Thromboprophylaxis in PC Patients

Since 2013, the ITAC CPGs have recommended the use of thromboprophylaxis with LMWH in surgical PC patients undergoing major surgery, hospitalized patients with acute medical illness and reduced mobility [12,13,14], and in locally advanced or metastatic ambulatory PC patients receiving chemotherapy [12,13,14]. New data have now emerged on the benefit of DOACs for primary thromboprophylaxis, which provide another option in selected patients [96,98]. The ITAC working group [14], the American Society of Clinical Oncology (ASCO) [15], and the National Comprehensive Cancer Network (NCCN) [109] updated their recommendations for VTE prophylaxis in cancer patients in 2019. 

### 5.1. Thromboprophylaxis in Surgical PC Patients

Thromboprophylaxis is recommend in PC patients undergoing major surgery by all current guidelines [12,13,14,15,109]. The 2019 ITAC CPGs [14] recommend thromboprophylaxis with the highest prophylactic dose of LMWH in PC patients undergoing major surgery, in the absence of contraindications (creatinine clearance <30 mL·min^−1^, high bleeding risk, active bleeding) [Grade 1A]. Low dose of unfractionated heparin (UFH) three times daily can also be used [Grade 1A]. There are insufficient data to support the use of fondaparinux as an alternative to LMWH in surgical PC patients [2C] and no data to support the use of DOACs [Best clinical practice]. Extended prophylaxis for 4 weeks should be used in patients undergoing laparotomy or laparoscopic surgery with a low bleeding risk [Grade 1A]. External compression devices are not recommended as monotherapy, except when pharmacological methods are contraindicated [Grade 2B], and the use of inferior vena cava filter is not recommended for routine thromboprophylaxis [Grade 1A] [14].

### 5.2. Thromboprophylaxis in Hospitalized PC Patients with Acute Medical Illness or with A Reduced Mobility

All current CPGs recommend thromboprophylaxis in hospitalized PC patients with acute medical illness or reduced mobility in the absence of bleeding or other contraindications [12,13,14,15,109]. The 2019 ITAC CPGs [14] recommend to use of prophylactic dose of LMWH in hospitalized PC patients with acute medical illness or with a reduced mobility in the absence of contraindications (creatinine clearance <30 mL·min^−1^, high bleeding risk, active bleeding) [Grade 1B]. Prophylaxis with UFH or fondaparinux can also be used [Grade 1B], but DOACs are not recommended routinely in this setting due to the lack of data [Best clinical practice] [14].

### 5.3. Thromboprophylaxis in Ambulatory PC Patients Receiving Chemotherapy

The KS assigns +2 points for PC patients. Therefore, according to the most recent guidelines, all ambulatory PC patients should be considered for thromboprophylaxis with either LMWH or DOACs. [14,15] In locally advanced or metastatic PC patients, the 2019 ITAC CPGs [14] recommend primary prophylaxis with LMWH for those patients having a low risk of bleeding and receiving systemic anticancer therapy [Grade 1B] [14], based on available evidence. [93,94,95] The 2019 ITAC CPGs [14] also recommend thromboprophylaxis with apixaban or rivaroxaban in cancer outpatients at intermediate-to-high risk (KS ≥2 prior to starting chemotherapy) with a low bleeding risk and in the absence of drug-drug interactions [Grade 1B] [14].

Similarly, the ASCO guidelines recommend that thromboprophylaxis with apixaban, rivaroxaban or LMWH may be offered in high-risk cancer outpatients (KS ≥2 or higher prior to starting a new systemic chemotherapy regimen) in the absence of significant risk factors for bleeding and drug interactions [15]. 

## 6. Conclusions

Evidence (Grade 1B) that appropriate use of primary thromboprophylaxis significantly and safely reduces the burden of VTE in PC patients has been available since 2013. Despite this fact, thromboprophylaxis remains largely underused. Increased awareness among healthcare professionals and adherence to evidence-based guidelines can decrease the burden of VTE in PC patients. Clinical tools based on the 2019 ITAC-CME international guidelines, such as a free accessible web-based mobile application with a decision-tree algorithm (downloadable at www.itaccme.com), can be used to assist clinicians in optimizing treatment in daily clinical practice. In the absence of head-to-head comparison between LMWH and DOACs, a discussion with the patient about the relative benefits and risks, drug cost, duration and tolerance of prophylaxis is warranted before prescribing thromboprophylaxis in PC ambulatory patients. 
